# 

**Published:** 1901-10-19

**Authors:** 


### NOTICE TO CORRESPONDENTS.

All MSS., Letters, Books for Review, and other matters intended for the Editor should be addressed The Editor, "The Hospital," The Hospital Building, 28 & 29 Southampton Street, Strand, W.C.
The Editor cannot undertake to return rejected MS., even when accompanied by stamped directed envelope.

**Notice to Advertisers and Subscribers.**

All Advertisements, Orders for Copies of The Hospital, and Business Communications should be addressed to **THE MANAGER (Not to the Editor)**, The Hospital, 28 & 29 Southampton Street, Strand, London, W.C.

The Cost of Subscription, post free, is as follows:—Per week, 2½d.; per quarter, 2s. 8d.; per half-year, 5s. 3d.; per annum, 10s. 6d. Foreign:—Per annum, 15s. 2d., payable, in all cases, in advance.

**NOTICE.—Vol. XXX. of "The Hospital" (April 1901, to September 1901), in handsome cloth binding will shortly be on sale at the Office of this Journal, price, 7s. 6d. Cases for binding the Numbers contained in this Volume 1s. 6d. Index to Vol. XXX., 2½d., post free.**

**The Monthly Part for October is now ready. Price 6d., by post 8d.**

### BOOKS RECEIVED.

Church Missionary House.

"Preaching and Healing."

Cassell and Co.

"Surgical Applied Anatomy." By Sir Frederick Treves, K.C.V.O., C.B., F.R.C.S. New Edition, revised with the assistance of Arthur Keith, M D., F.R C.S.

"Advice to Women." By Florence Stacpoole.

Baillière, Tindall, and Cox.

"Syphilis and other Venereal Diseases." By H. de Meric.

Messrs. Longmans, Green and Co.

"Anatomy, Descriptive and Surgical." By Henry Gray, F.R.S. Edited by T. Pickering Pick, F.R.C.S., and Robert Howden, M.A., M.B., C.M.

The National Antivivisection Society.

"Dr. Andrew Wilson's Logic."

H. K. Lewis.

"Reports of the Medical, Surgical, and Pathological Registrars of the Middlesex Hospital for 1899."

### Scraps and Gleanings.

The supply of hops from Kent this year has fallen from 400,000 cwt. to about 330,000 cwt.

Since the foundation of Hospital Saturday £305,809 has been contributed to the medical charities through its agency.

The "London Hospital" Lodge was consecrated recently by the Grand Secretary. This is the fourth lodge in London connected with metropolitan hospitals.

The gross cost of the relief of the poor in the Metropolis during the year ended Lady Day, 1900, was £11,567,649, which is greater than that recorded in any previous year.

The Committee of Management of the North London Hospital for Consumption have selected the design of Mr. Frederick Wheeler, F.R.I.B.A., for the proposed country branch and convalescent home at Northwood.

The new "Rob Roy" Home for Cripples, at Margate, which was recently opened by the Marquis of Northampton, contains accommodation for 30 boys. This home is carried on by the Ragged School Union.

The number of paupers in receipt of relief in the Metropolis on January 1st, 1901, amounted to 801,347, a decrease of 164 as compared with the preceding January. Of these 96,277 are returned as insane, including 1,726 insane children.

Last year nearly 600 inebriates capable of paying from 15s. to 30s. a week made application to the Home Office for particulars of a home where they could enter. Such accommodation was, however, then lacking, but the Salvation Army is on the eve of opening such a home for the reception of male inebriates, where about 40 persons will be accommodated.

### The Student of Medicine.

### Appointments.

**Cyril P. Burd**, M R.C.S.Eng., L.R.C.P.Lond., appointed House Surgeon to the Salop Infirmary, Shrewsbury. **G. Calthorp**, M.B., B.C.Cantab., appointed District Medical Officer of the Walsingham Union. **E. Tenison Collins**, M.R.C.S.Eng., appointed Second Gynæcologist at the Cardiff Infirmary. **Arthur Cutfield**, B.A.Cantab., M.R.C.S.Eng., appointed Medical Officer of Health to the Ross Urban District Council. **William C. Ellis**, L.R.C.P. and S.Edin., etc., appointed Deputy Medical Officer of Health to the County Borough of Brighton, and House Physician to the Borough Sanatorium. **G. A. Finlayson**, M.B., Ch.B.Aberd., appointed House Physician at the Great Northern Central Hospital. **C. Hadley**, M.R.C.S.Eng., L.R.C.P., appointed District Medical Officer of the Foleshill Union. **Ernest Hill**, L.R.C.P., M.R.C.S., D.P.H., appointed Health Officer for the Colony of Natal. **John Howell**, M.B., B.S.Lond., F.R.C.S.Eng., appointed Honorary Surgeon to the General Hospital, Cheltenham. **M. F. Krnna**, L.R.C.P., L.R.C.S.Irel., appointed Coroner for South Kildare. **J. M. A. Lamb**, L.S.A.Lond., appointed District Medical Officer and Public Vaccinator to the Second District of the Poole Union. **Samuel Montgomery**, L.R.C.P., L.R.C S.Ed., appointed District Medical Officer and Public Vaccinator to the Branksome District of the Poole Union. **Denis Murphy**, L.R.C.P., L.R.C.S.Edin., appointed Pathologist to the North Charitable Infirmary, Cork. **Wm. Paynter Noall**, M.B.Lond., M.R.C.S.Eng., L.R C.P.Lond., appointed House Surgeon to the East London Hospital for Children. **Claud M. Pennefather**, M.R.C.S., L.R.C.P., M.B., B.S.Durh., appointed Junior House Physician to the Great Northern Central Hospital. **Arthur Y. Pringle**, M.R.C.S.Eng., L.R.C.P.Lond., appointed Honorary Medical and Surgical Officer to the East Suffolk and Ipswich Hospital. **James Sherren**, F.R.C.S.Eng., appointed Surgical Registrar to the London Hospital. **W. R. Speirs**, M.B.Glasg., C.M., appointed Medical Officer of Health for the Haltwhistle Rural District. **W. Heywood Waddington**, M.B., B.Ch.Vict., appointed Medical Officer of Health for the Borough of Scarborough. **R. A. Walker**, B.A., B.C.Cantab., M.R.C.S., L.R.C.P., appointed Junior House Surgeon to the Great Northern Central Hospital. **William Henry Whitehouse**, M.D.Durh., D.P.H.Edin., appointed Honorary Surgeon to the Birmingham Lying-in Charity. **Leonard Williams**, M.D.Glas., appointed Assistant Physician to the German Hospital, Dalston, N.

